# A Novel Recognition Strategy for Epilepsy EEG Signals Based on Conditional Entropy of Ordinal Patterns

**DOI:** 10.3390/e22101092

**Published:** 2020-09-29

**Authors:** Xian Liu, Zhuang Fu

**Affiliations:** Key Laboratory of Intelligent Rehabilitation and Neuromodulation of Hebei Province, Yanshan University, Qinhuangdao 066004, China; zhuangfu126@126.com

**Keywords:** epileptic seizure, electroencephalogram (EEG), conditional entropy of ordinal patterns (CEOP), neural mass model, recognition

## Abstract

Epilepsy is one of the most ordinary neuropathic illnesses, and electroencephalogram (EEG) is the essential method for recording various brain rhythm activities due to its high temporal resolution. The conditional entropy of ordinal patterns (CEOP) is known to be fast and easy to implement, which can effectively measure the irregularity of the physiological signals. The present work aims to apply the CEOP to analyze the complexity characteristics of the EEG signals and recognize the epilepsy EEG signals. We discuss the parameter selection and the performance analysis of the CEOP based on the neural mass model. The CEOP is applied to the real EEG database of Bonn epilepsy for identification. The results show that the CEOP is an excellent metrics for the analysis and recognition of epileptic EEG signals. The differences of the CEOP in normal and epileptic brain states suggest that the CEOP could be a judgment tool for the diagnosis of the epileptic seizure.

## 1. Introduction

The diagnosis of neurological diseases has always been difficult and challenging problems in the biomedical field. As we have known, epilepsy is one of the most ordinary neuropathic illnesses. Epileptic seizure makes many serious consequences about the patients’ physiology and psychology in their daily lives and disrupts the normal cognition, consciousness, sleep, emotion, etc. [1,2]. Abnormal firing of neurons in different parts of the brain results in a variety of biological manifestations, and epilepsy is caused by disordered excessive or hyper-synchronous neuronal activity, which is recurrent and unprovoked seizures [3,4,5]. Thus, it is necessary to understand the pathogenesis mechanism of various neurological and mental diseases. Computational models deliver a potential means for offering an explanation for brain activities and disorders by mathematical modeling. It improves our understanding of abnormal brain electrical activities [6]. After many years, the computational neural model has derived a variety of variations and it has been well used in [7,8,9]. We employ the widely used neural mass model with nonlinear lumped parameters that can simulate various physiological signals in this work [10]. This mesoscopic model provides several interconnected neural masses that are characterized by a handful of state variables to interpret plentiful complex physiological and pathological phenomena.

EEG plays a crucial and significant role in the diagnosis of epilepsy [11,12]. However, long-term visual examination is time-consuming and considerably laborious, and it may produce unnecessary human empirical error. Thus, recognizing epileptic electroencephalogram (EEG) automatically and effectively is urgent and vital research. Recent studies have shown various automatic seizure prediction and distinction methodologies. Polat and G*ü*nes performed the classification of epileptiform EEG while using a hybrid system that was based on decision tree classifier and Fast Fourier Transform (FFT), and it obtained great classification accuracy [13]. Vézard and Legrand used common spatial pattern (CSP) combined with linear discriminant analysis (LDA) to establish a decision structure to predict the subjects’ alertness [14]. Yuan and Zhou developed an approach to detect seizures employing log-Euclidean Gaussian kernel-based sparse representation (SR) in long-term EEG recordings [15]. Seo and Tsuda proposed a new method that was based on the dynamic mode decomposition (DMD) in order to find a distinctive contrast between the ictal and inter-ictal patterns [16]. When compared with previous time-domain analysis, frequency-domain analysis, and time-frequency analysis [17,18], the entropy based nonlinear analysis method has been applied to characterize brain activities to research the pathophysiological mechanisms underlying the neurological conditions [19,20]. Entropy is a metrics which is different from fractal dimension, and it is a kind of index to measure the probability of new pattern in nonlinear time series. Using the entropy measure effectively and obtaining the corresponding entropy index can help us better analyze and understand the complex and interesting brain activities. Echegoyen and López-Sanz studied the differences between permutation entropy (PE) and statistical complexity (SC) in broadband signals and the decomposition into frequency bands, showing that SC does not necessarily decrease in Alzheimer’s Disease (AD) [21]. Harezlak and Kasprowski used fuzzy entropy (FE) in order to reveal eye movement signal characteristics, and this classification produced an improvement in the accuracy for saccadic latency and saccade [22]. Hussain and Wang put forward a new entropy index of permutation fuzzy entropy (PFEN), which may delineate the epileptic seizure between ictal and inter-ictal state while using different machine learning classifiers [23]. Nicolini and Forcellini used a novel information-theoretic approach based on Von-Neumann entropy. They provided a measure of information encoded in the networks at different scales and defined a measure of distance between networks [24]. Increasing research results show that entropy measurement is successful and effective in the analysis and recognition of EEG signals, which encourages us to focus on exploring more methods to be applied in practice and study more brain function, so as to better serve society in health care.

In our previous works, we successfully applied the modified permutation entropy (MPE) and approximate entropy (AE) to the analysis of EEG signals, not only provided a new method for epilepsy detection and quantitative analysis [25], but also applied the entropy algorithm to the modulation process of the abnormal brain rhythm [26]. Now, we concentrate on the conditional entropy of ordinal patterns (CEOP) combined with the support vector machine (SVM) in order to analyze and recognize the epileptic EEG signals. Unakafov and Keller first proposed the CEOP [27], and the ordinal pattern based algorithm is easy and fast to implement. It can overcome the high computational cost [28]. In the latest research, Unakafov and Keller introduced the statistic based on CEOP that characterized the local up and down in the time series, and the proposed method did not detect pure level changes, but rather changes in the intrinsic pattern structure of the time series [29]. Mougoufan and Fouda creatively employed CEOP to the detection of abnormal ECG beats and achieved good results [30]. Rubega and Scarpa used the CEOP with other metrics in order to assess the complexity in Euglycemia and Hypoglycemia, and gained the accurate classification of the glycemic state through EEG data [31]. In this work, we also successfully fulfill the analysis and recognition of the epilepsy EEG signals based on the CEOP which provides a basis for us to study the mechanisms of the brain diseases.

The rest of the paper is organized, as follows: Section 2 presents an overview about the Bonn epilepsy EEG database and the neural mass model. The Bonn epilepsy EEG database is commonly used in recognition of the epilepsy EEG signals, and the neural mass model can simulate various EEG signals by setting different parameters. Section 3 introduces the overall scheme and the methods used. Section 4 is the main related results. We introduce the influence of various parameters on the CEOP with the help of the neural mass model. In addition, the distinguishing ability of the CEOP under different epileptic intensity and noise intensity is discussed. We also make a comparison between the CEOP and some existing entropy algorithms, like PE and MPE. Subsequently, we employ the Bonn epilepsy EEG database in order to verify the proposed approach. Section 5 gives some discussions of the proposed scheme, and the advantages and limitations of the proposed scheme are discussed in combination with the experimental results. Finally, Section 6 gives some concluding remarks.

## 2. Materials

### 2.1. Bonn Epilepsy EEG Database

The Bonn epilepsy database is maintained by the Department of Epileptology, University of Bonn, which includes five data sets, denoted by A-Z, B-O, C-N, D-F, and E-S. Each data set is composed of 100 single-channel scalp or intracranial EEG with sampling frequency 173.61 Hz and 12-bit A-D resolution. Table 1 summarizes details of the Bonn epilepsy database.

### 2.2. Neural Mass Model

**Figure 1:**
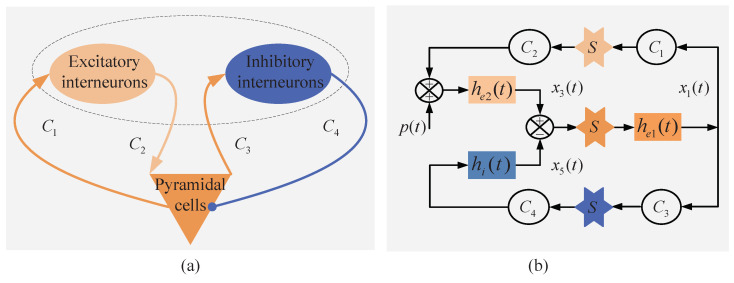
The structure and block diagram of the neural mass model: (**a**) the simplified structure of the neural mass model. (**b**) The block diagram of the neural mass model.

We consider to adopt it in the analysis of the epileptic EEG signals based on the advantages of the neural mass model to simulate brain activities. The single neural mass model presented in Figure 1 is constitutive of the pyramidal cells and interneurons. The pyramidal cells receive excitatory feedback $h_{e2}\left(t\right)$ and inhibitory feedback $h_{i}\left(t\right)$ from the interneurons. The interneurons only receive the excitatory inputs $h_{e1}\left(t\right)$. The impulse response of the linear transfer function is given by:

(1) $$\begin{array}{c} h_{em}\left(t\right)=\xi \left(t\right)Aate^{-at},m=1,2.\\ h_{i}\left(t\right)=\xi \left(t\right)Bbte^{-bt} \end{array}$$

where ξ(t) is the Heaviside function. Six first-order ordinary differential equations can describe the single neural mass model:

(2) $$\left\{\begin{array}{c} {\dot{x}}_{1}\left(t\right)=x_{2}\left(t\right)\\ {\dot{x}}_{2}\left(t\right)=AaS\left(x_{3}\left(t\right)-x_{5}\left(t\right)\right)-2ax_{2}\left(t\right)-a^{2}x_{1}\left(t\right)\\ {\dot{x}}_{3}\left(t\right)=x_{4}\left(t\right)\\ {\dot{x}}_{4}\left(t\right)=Aa\left[p\left(t\right)+C_{2}S\left(C_{1}x_{1}\left(t\right)\right)\right]-2ax_{4}\left(t\right)-a^{2}x_{3}\left(t\right)\\ {\dot{x}}_{5}\left(t\right)=x_{6}\left(t\right)\\ {\dot{x}}_{6}\left(t\right)=BbC_{4}S\left(C_{3}x_{1}\left(t\right)\right)-2bx_{6}\left(t\right)-b^{2}x_{5}\left(t\right) \end{array}\right.$$

The variables $x_{1}\left(t\right)$, $x_{3}\left(t\right)$, and $x_{5}\left(t\right)$ are the outputs of different types of postsynaptic potential blocks, while $x_{2}\left(t\right)$, $x_{4}\left(t\right)$, and $x_{6}\left(t\right)$ are the time derivatives of $x_{1}\left(t\right)$, $x_{3}\left(t\right)$, and $x_{5}\left(t\right)$, respectively. The parameters *A* and *B* are average excitatory and inhibitory synaptic gains that determine the maximal amplitude of the postsynaptic potentials. The parameter *a* is the membrane average time constant, and *b* is the average distributed time delays in the dendritic tree. The constants $C_{1}$, $C_{2}$, $C_{3}$, and $C_{4}$ account for the intrinsic circuitry and average number of synaptic contacts, which summarize the interactions between the pyramidal cells and the interneurons. The input p(t) is the afferent influence from neighbouring or more distant populations. It is represented by a pulse density which can be any arbitrary function including white noise [10,32,33]. The static nonlinear transfer function S(v) has the sigmoid form:

(3) $$S\left(v\right)=\frac{2e_{0}}{1+e^{r(v_{0}-v)}}$$

where $2e_{0}$ is the maximum firing rate, $v_{0}$ is the postsynaptic potential corresponding to a firing rate $e_{0}$, and *r* is the steepness of the sigmoid. The output of the neural mass model is defined as:

(4) $$y\left(t\right)=x_{3}\left(t\right)-x_{5}\left(t\right)$$

All of the parameters are set on a physiological basis, and the setting of them was summarized in [10,32,33]. The standard values are listed, as follows.

(5) $$\begin{array}{c} A=3.25\;\mathrm{mV},B=22\;\mathrm{mV},a=100\;s^{-1},b=50\;s^{-1},\\ C_{1}=135,C_{2}=108,C_{3}=33.75,C_{4}=33.75,\\ v_{0}=6\;\mathrm{mV},e_{0}=2.5\;s^{-1},r=0.56\;{\mathrm{mV}}^{-1}. \end{array}$$

The model with the standard parameters can produce well-defined alpha rhythms in the EEG signals. The alteration of some key parameters, like *A*, may result in the epileptiform spikes in the EEG signals. Increasing *A* leads to the aberrant outputs that resemble the real EEG signals that are related to some neuropsychiatric disorders [10]. The neural mass model produces the inter-ictal EEG signals when A=3.4 mV and the ictal EEG signals as *A* increases to more than 3.4 mV. We set the excitability gain parameter A=3.8 mV for the sake of research.

## 3. Scheme and Methods

### 3.1. Conditional Entropy of Ordinal Patterns

**Figure 2:**
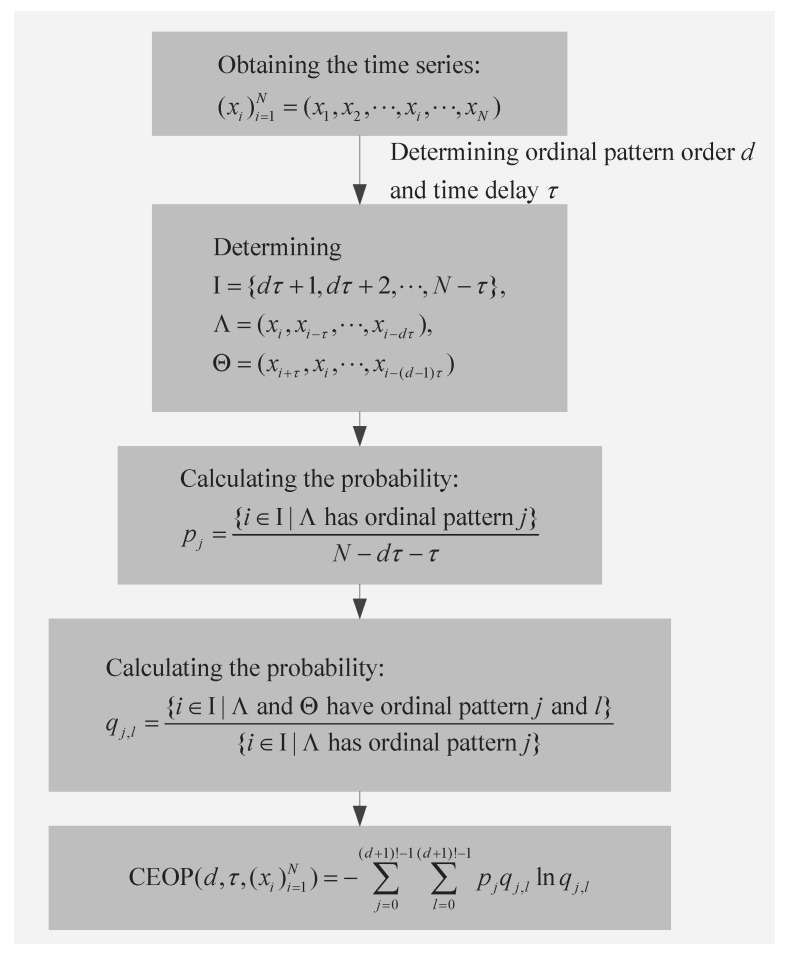
The flow chart of the conditional entropy of ordinal patterns.

Ordinal pattern based algorithms are used to measure the complexity in time series broadly and effectively, and the ordinal pattern is the basis of the conditional entropy. The CEOP takes the order relation between samples of the time series into account instead of the values themselves. It characterizes the diversity of successors of a given ordinal pattern, whereas other algorithms always characterize the diversity of ordinal patterns themselves. Thus, it is obvious that the CEOP characterizes the average diversity of the ordinal patterns. The CEOP can not only measure the irregularity of the signals effectively, but it also has good anti-noise and anti-interference ability [27]. Figure 2 illustrates the flow chart of the conditional entropy of ordinal patterns, and the process is as follows.

(1) Given the time series:

(6) $${\left(x_{i}\right)}_{i=1}^{N}=\left(x_{1},x_{2},\cdots ,x_{i},\cdots ,x_{N}\right)$$

and determine the ordinal pattern order *d* and the time delay τ. The ordinal pattern order *d* may be 3, 4, 5, 6, and 7, as shown in [34,35,36]. The time delay τ between the successive points is selected as 1 $s^{-1}$ empirically. We will discuss the effects of different ordinal pattern order *d* and time delay τ on the CEOP that is based on the neural mass model in Section 4.

(2) Calculate the probability of pairs of ordinal patterns $p_{j}$ and $q_{j,l}$, and the ordinal pattern *l* occurs after the ordinal pattern *j*.

(7) $$p_{j}=\frac{\left\{i\in I\;|\;\Lambda \;\mathrm{has}\;\mathrm{ordinal}\;\mathrm{pattern}\;j\right\}}{N-d\tau -\tau }$$

(8) $$q_{j,l}=\frac{\left\{i\in I\;|\;\Lambda \;and\;\Theta \;\mathrm{have}\;\mathrm{ordinal}\;\mathrm{pattern}\;j\;and\;l\right\}}{\left\{i\in I\;|\;\Lambda \;\mathrm{has}\;\mathrm{ordinal}\;\mathrm{pattern}\;j\right\}}$$

for

(9) $$I=\left\{d\tau +1,d\tau +2,\cdots ,N-\tau \right\}$$

and

(10) $$\Lambda =(x_{i},x_{i-\tau },\cdots ,x_{i-d\tau }),\;\Theta =(x_{i+\tau },x_{i},\cdots ,x_{i-(d-1)\tau })$$

(3) Calculate the CEOP of ordinal pattern order *d* and time delay τ.

(11) $$\mathrm{CEOP}\left(d,\tau ,{\left(x_{i}\right)}_{i=1}^{N}\right)=-\sum_{j=0}^{(d+1)!-1}\sum_{l=0}^{(d+1)!-1}p_{j}q_{j,l}\ln\left(p_{j}q_{j,l}\right)+\sum_{j=0}^{(d+1)!-1}p_{j}\ln p_{j}$$

The negative log of the probability represents the amount of information that is carried by a possible event. Multiply the amount of information possible by the probability that it is going to happen, then sum them up. The formula (11) can be simplified, as follows.

(12) $$\mathrm{CEOP}\left(d,\tau ,{\left(x_{i}\right)}_{i=1}^{N}\right)=-\sum_{j=0}^{(d+1)!-1}\sum_{l=0}^{(d+1)!-1}p_{j}q_{j,l}\ln q_{j,l}$$

The more complex the system is, the more different kinds of situations there are, and the higher entropy. On the contrary, the simpler the system, the smaller the number of situations, and the lower the entropy will be [27,28,29]. In the extreme cases, there is only one case where the entropy is zero. Intuitively, extremely large entropy means that the system is infinitely complex and chaotic. Indeed, the higher the diversity of ordinal patterns of order *d* in the time series, the larger the value of the CEOP. According to [28], the range of values of the CEOP is as follows.

(13) $$0\leq \mathrm{CEOP}\left(d,\tau ,{\left(x_{i}\right)}_{i=1}^{N}\right)\leq \ln\left(d+1\right)$$

In other words, the larger order *d*, the better estimation of complexity underlying the system by the CEOP. However, excessively high *d* may lead to an underestimation of the complexity of the system. In the finite length range of the time series, not all ordinal patterns that respresent the system can occur. In [28], the relationship between *N* and *d* is given, as follows.

(14) (d+1)(d+1)!<N

### 3.2. Variation Coefficient

In order to be able to clearly see the difference of the CEOP between the normal state and epileptic state, we define η in order to represent the difference, and the variation coefficient η can be defined as:

(15) $$\eta =\frac{\left|{\mathrm{CEOP}}_{\mathrm{normal}}-{\mathrm{CEOP}}_{\mathrm{seizure}}\right|}{{\mathrm{CEOP}}_{\mathrm{normal}}}$$

where ${\mathrm{CEOP}}_{\mathrm{normal}}$ is the entropy value of the normal state and ${\mathrm{CEOP}}_{\mathrm{seizure}}$ is the entropy value of the epileptic state. We set the excitability gain parameter *A* to 3.25 mV and 3.8 mV for the normal state and the epileptic state, as shown in Section 2.2. The η is affected by the algorithm parameters, including ordinal pattern order *d* and time delay τ, and the model parameters including excitability gain parameter *A* and input Gaussian white noise p(t). We will discuss the change law on the variation coefficient η by setting different parameters in Section 4. The η is between 0 and 1; the larger its value, the more obvious the difference between the normal state and epileptic state, and the better recognition effect of the CEOP.

### 3.3. k-Fold Cross-Validation

The *k*-fold cross-validation is a statistical method for eliminating the training bias that is caused by the sampling randomness. It can extract as much useful information as possible from the limited data. Thus, we consider adopting it to evaluate the classification performance of training the SVM classifier. The process of the *k*-fold cross-validation is summarized, as follows.

(1) The data set Φ is divided into *k* disjoint subsets of the same size. The corresponding subsets are denoted as $\left\{\Phi_{1},\Phi_{2},\cdots ,\Phi_{k}\right\}$. The number of samples in Φ is *m*. Each subset has $\frac{m}{k}$ samples.

(2) Select $\Phi_{i}$ as the testing set in the subsets, and $\left\{\Phi_{1},\cdots ,\Phi_{i-1},\Phi_{i+1},\cdots ,\Phi_{k}\right\}$ are the training sets. The experiment is carried out in order to obtain the corresponding accuracy of the classification.

(3) After a total of *k* experiments, the average accuracy of the classification is obtained. The higher the average accuracy, the better the classification performance.

We choose 10-fold cross-validation in this work.

### 3.4. Evaluation Index

In a typical binary classification problem, suppose there are a set of test samples that fall into only two categories: positive and negative. The predicted value is positive, which is P (Positive) and the predicted value is negative, which is N (Negative). The predicted value is the same as the true value, which is T (True) and the predicted value is contrary to the true value, which is F (False). The representations are summarized in Table 2. The total number of true positive examples in the sample is TP+FN, and the TPR is the true positive rate as TP/(TP+FN). Similarly, the total number of true counterexample categories in the sample is TN+FP and the FPR is the false positive rate as FP/(TN+FP). The Receiver Operating Characteristic (ROC) curve is the drawing of the corresponding TPR and FPR that resulted in the two-dimensional coordinate system, where the horizontal axis is represented by FPR. The AUC (Area Under ROC Curve) value is the area that is covered by the ROC curve, and the larger the AUC, the better the classification effect. In addition, other evaluation indexes are defined, as follows.

(16) $$\mathrm{Sensitivity}=\frac{\mathrm{TP}}{\mathrm{TP}+\mathrm{FN}}$$

(17) $$\mathrm{Specificity}=\frac{\mathrm{TN}}{\mathrm{TP}+\mathrm{FN}}$$

(18) $$\mathrm{Accuracy}=\frac{\mathrm{TP}+\mathrm{TN}}{\mathrm{TP}+\mathrm{TN}+\mathrm{FP}+\mathrm{FN}}$$

Sensitivity is the number of true positives/the total number of ictal EEG epochs that are labeled by the EEG specialists, and true positive represents the ictal EEG identified by algorithm and experts. Specificity is the number of true negatives/the total number of inter-ictal EEG epochs that are labeled by the EEG specialists, and true negative represents the inter-ictal EEG identified by algorithm and experts. Accuracy is the number of correctly identified epochs/the total number of epochs.

### 3.5. Overall Scheme

**Figure 3:**
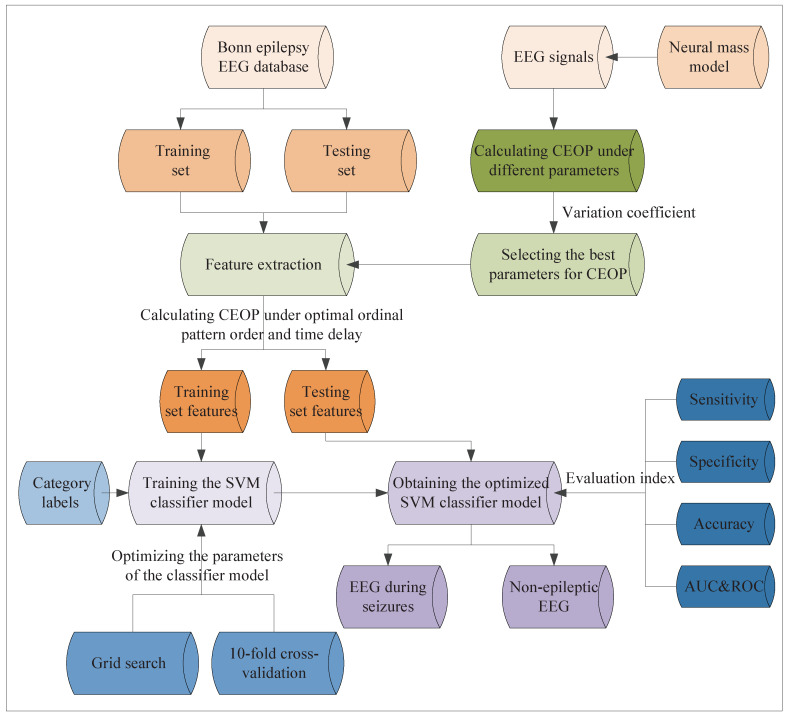
The scheme of the recognition of the Bonn epilepsy EEG database based on the conditional entropy of ordinal patterns (CEOP).

This part is mainly to give the overall research scheme, and the scheme of the recognition of the Bonn epilepsy EEG database that is based on the CEOP is shown in Figure 3. The main idea is to select the CEOP as the eigenvector in feature extraction, then feed it to the SVM classifier in order to recognize the epileptic EEG signals. The optimal parameters of calculating the CEOP are obtained by means of the analysis of the neural mass model and the variation coefficient. Grid search and 10-fold cross-validation determine the optimal parameters of SVM, and then the optimal SVM classifier model is obtained. Section 4 will provide the division of the data sets, the processes of the feature extraction and classification, and the results of specific evaluation index.

Based on the unique advantages of the support vector machine (SVM) in solving the pattern recognition with small sample and high dimension, we consider applying it to the classification of the epilepsy EEG signals [37]. Penalty factor *c* is a key parameter in SVM and it plays a role in balancing the generalization ability and fitting ability of the model. The SVM classifier can address the linear or nonlinear classification problems by setting different kernel functions. In this work, we use the radial basis function (RBF) as the kernel function of the SVM classifier. *g* is the width parameter of the RBF, and it mainly affects the complexity of the distribution of sample data in the high-dimensional feature space [38]. The calibration of the penalty factor *c*, the width parameter of the RBF *g*, and the training bias that is caused by the small sample are the difficulties in the practical application of SVM. In this paper, the grid search is used in order to optimize the parameter pair (c,g) simultaneously, which avoids the local optimization. The 10-fold cross-validation is used to evaluate the training performance of the SVM classifier model. In the subsequent experiments, these are accomplished using the toolkit libsvm on MATLAB 2015a. The optimization of the parameters of the SVM classifier based on 10-fold cross-validation and grid search is concluded in Algorithm 1.
**Algorithm 1:** Optimizing the parameters of the Support vector machine (SVM) classifier based on 10-fold cross-validation and grid search.
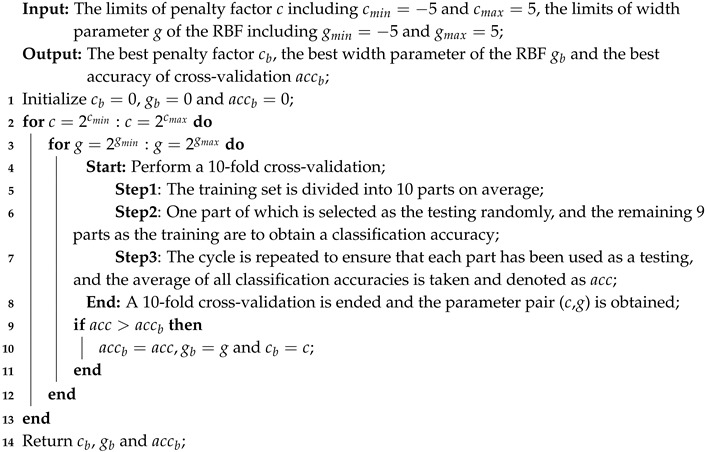


## 4. Results

We use the CEOP combined with the neural mass model in order to discuss the different traits from normal state, inter-ictal state to ictal state. The model is used to simulate the non-epileptic and epileptic EEG signals by setting different excitability gain parameter *A* (A=3.25 mV, A=3.4 mV and A=3.8 mV) in this work. The mean and variance (corresponding to a rate of 30–150 pulses/s) of the p(t) are adjusted so that the neural mass model can produce the signals that are similar to the spontaneous EEG recorded from neocortical structure electrodes, thus p(t) is modeled by a Gaussian white noise with mean value 101 and standard deviation 35 in this work [32,39]. The higher mean value means greater noise intensity. We keep the rest of the parameters standard. The ordinary differential equations (2) are solved by using the fourth-order Runge–Kutta method with time step 0.005 for 30,000 time steps. In addition, our experiment on the CEOP is aided by the OPA (ordinal-patterns-analysis) toolbox in MATLAB 2015a in this work, and the OPA toolbox is intended for the nonlinear analysis of multivariate time series, such as the EEG signals. It becomes increasingly widely used in ordinal-patterns-based measures, which is easily computed and visualized.

**Figure 4:**
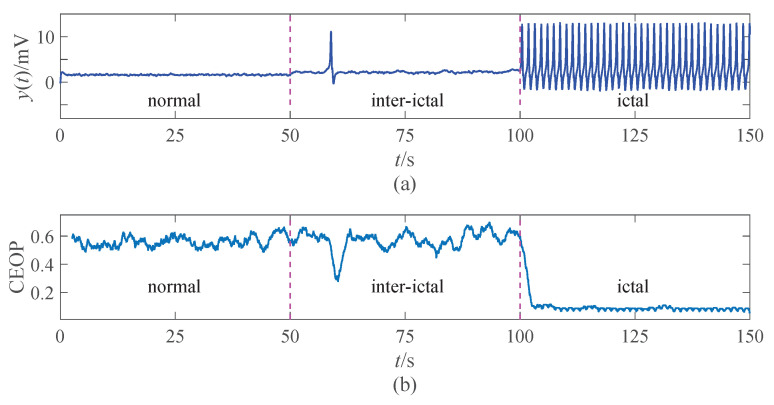
The output of the model by setting different excitability gain parameters *A*, and the corresponding values of the CEOP: (**a**) The waveforms in three states. (**b**) The values of the CEOP in three states.

The results presented in Figure 4 give an intuition that the CEOP between the normal state (A=3.25 mV) and the inter-ictal state (A=3.4 mV) is similar, both of which are relatively high, especially when there is a decrease in sporadic spikes. The CEOP during the ictal state (A=3.8 mV) is lower, which can be used as a feature for distinguishing the epileptic EEG signals. We will discuss the reasons for this phenomenon and give some reasonable explanations in the next parts.

### 4.1. Parameters Selection of the CEOP

#### 4.1.1. Ordinal Pattern Order *d*

**Figure 5:**
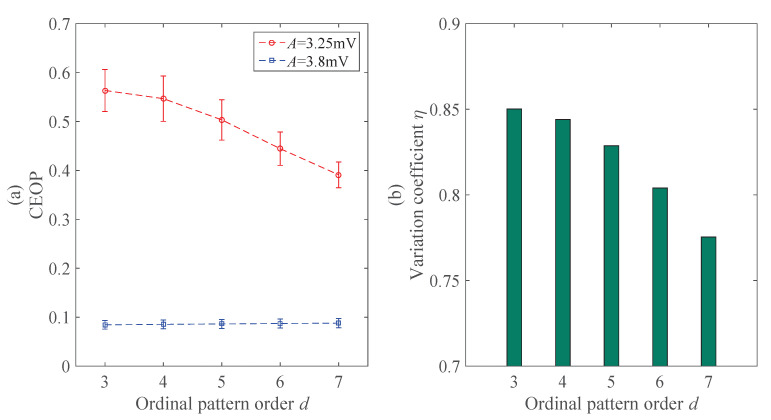
The effects of the ordinal pattern order *d* on the CEOP and the variation coefficient η: (**a**) The error bars of the CEOP under different ordinal pattern order *d*. (**b**) The law of change in the variation coefficient η.

In this part, we mainly illustrate the effect of the ordinal pattern order *d* on the CEOP, and then choose the $d_{optimal}$. The constant before simulation is set as the time delay τ=1 $s^{-1}$, and others are standard values. Figure 5 illustrates that the CEOP gradually decreases in the epileptic state with the increase of *d*, but the CEOP is basically the same in the normal state, and the variation coefficient η decreases. The change can be seen, but it is not very strong; this shows that the sensitivity of the ordinal pattern order *d* on CEOP is small, and the change of *d* does not affect the accuracy of the recognition strongly. Although *d* has little sensitivity and there is no strong impact on the accuracy, the increase of *d* will increase the calculation cost and complicate the recognition processes. When considering this layer, it is reasonable to choose $d_{optimal}=3$ and apply it to the subsequent recognition experiment of the epileptic EEG signals.

#### 4.1.2. Time Delay τ

**Figure 6:**
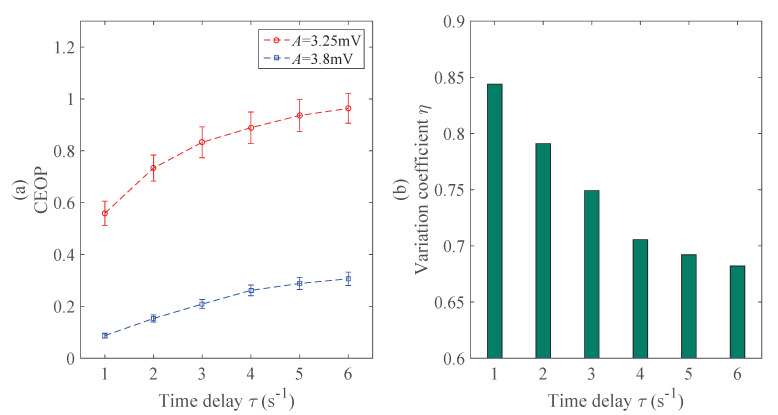
The effects of the time delay τ on the CEOP and the variation coefficient η: (**a**) The error bars of the CEOP under different time delay τ. (**b**) The law of change in the variation coefficient η.

In this part, we mainly introduce the effect of the time delay τ on the CEOP, and then choose the $\tau_{optimal}$. Previous studies have mostly chosen τ based on the experience, and we hope to illustrate the influence of the selection of τ on the CEOP by means of the neural mass model. The constant before simulation is set as the ordinal pattern order d=3 and others are standard values. The τ describes the time delay between the successive points in the symbol sequences. Figure 6 shows that, with the increase of τ, the CEOP increases in the epileptic state and normal state, but, in the case of τ=1 $s^{-1}$, the CEOP has the best discriminating ability in Figure 6b. Thus, $\tau_{optimal}$ is set as 1 $s^{-1}$, which is applied to the subsequent recognition experiment of the epileptic EEG signals.

To sum up, we examined two parameters that are important for the CEOP, and those are the ordinal pattern order *d* and the time delay τ. Here, we did not consider the sample size *N*. The early research [35] has emphasized that the length of the sequences are determined then the number of possible permutation patterns are determined. In our work, both *N* and *d* follow formula (14), and then we can only discuss one parameter, which is the ordinal pattern order *d*, which is more intuitive and of research significance. Both [35,36] discussed the influence of the selection of the parameters on the analysis and recognition of the EEG signals, including *d* and τ, and the ordinal pattern order *d* may be 3, 4, 5, 6, and 7, which were with little difference. And the τ was supposed to be selected was 1 $s^{-1}$. We gain the conclusion about *d* and τ to be proved basically as the same as [35,36]. However, instead of theoretical derivation and proof, they conducted a comprehensive survey of previous studies then led to the conclusion. It is a little bit more persuasive to determine the parameters that are based on the neural mass model with the help of variation coefficient η. In Figure 5b, the results show that the effect is slightly better than the others when d=3, and may reduce the corresponding calculation cost and simplify the experimental procedures. Thus, we will employ $d_{optimal}=3$ and $\tau_{optimal}=1$ $s^{-1}$ to do the following research.

### 4.2. Performances Analysis of the CEOP

We also design the comparison experiments and do a performance analysis about the PE, MPE, and CEOP, in order to facilitate the analysis and understanding of the effectiveness of the CEOP. These three algorithms are of the same type, and the results can effectively illustrate the advantages of the CEOP.

#### 4.2.1. The Analysis Result of Signals under Different Excitability Gain Parameter *A*

In this part, we mainly illustrate the effects of the seizure intensity by means of the excitability gain parameter *A*, and the constants before simulation are set as the ordinal pattern order $d_{optimal}=3$ and the time delay $\tau_{optimal}=1$ $s^{-1}$, and others are standard values.

**Figure 7:**
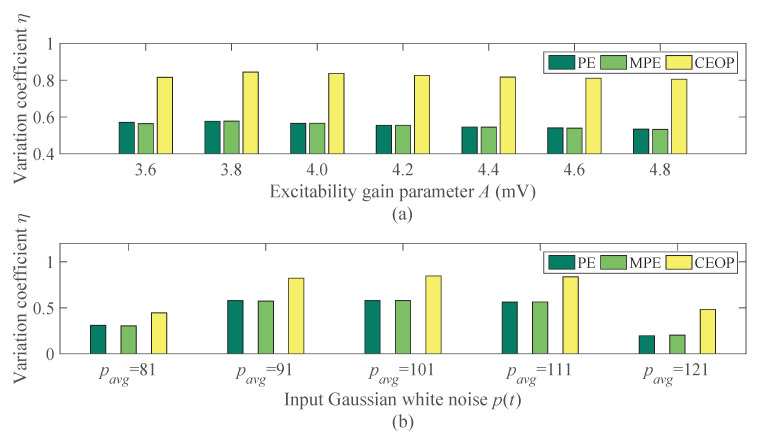
The law of change in the variation coefficient η under different seizure intensity and noise intensity: (**a**) The effects of the excitability gain parameters *A* on the variation coefficient η. (**b**) The effects of the input Gaussian white noise p(t) on the variation coefficient η.

Table 3 illustrates the mean and standard deviation of the entropy value under different excitability gain parameters *A*. The three entropies are similar in the normal and epileptic state. All of the three are relatively high in the normal state and low in the epileptic state. The entropy tends to be stable in the epileptic state and it does not change with the increase of the seizure intensity. Due to the relatively low amplitude and high oscillation frequency of the normal EEG signals the entropy is higher, which indicates that the time series are the most complex. The continuous discharge during epileptic seizures has relatively high amplitude and low oscillation frequency, and the entropy is lower, indicating that there are the most regular time series. The complexity of the continuous discharge in the epileptic state is significantly lower than the normal EEG signals and the sporadic spikes of the inter-ictal state, so it is easy to distinguish the epilepsy EEG signals. The mean value of the CEOP is less than the PE and MPE in Table 3, and the CEOP makes the best distinction performance in Figure 7a. With the increase of the *A*, the seizures are becoming increasingly serious, and it is clear that the effect of the recognition of the CEOP is always the best of the three.

#### 4.2.2. The Analysis Result of Signals under Different Input Gaussian White Noise p(t)

In this part, we mainly introduce the effects of the noise intensity by means of the input Gaussian white noise p(t), and the constants before simulation are set as the ordinal pattern order $d_{optimal}=3$, and the time delay $\tau_{optimal}=1$ $s^{-1}$, and others are standard values.

Table 4 shows the mean and the standard deviation of the entropy value under different input Gaussian white noise p(t) in the normal and epileptic state. The p(t), which is simulated by the Gaussian white noise, represents excitatory effects from adjacent or distant clusters of the cells in the neural mass model. The increase of the p(t) exacerbates this effect, which increases the excitability of the neural population. With the increase of the noise intensity, all three entropy values decrease, but finally tend to be stable, indicating that there is a continuous discharge in the epileptic state. Figure 7b illustrates that the anti-noise capability of the CEOP is the best among the algorithms of the same type, there is no denying that this ability will decrease slightly with the increase of the noise intensity. In the process of measuring bioelectrical signals, many noises will be mixed in the results due to the interference of instruments and human beings. The application of the CEOP can deal with these problems well.

### 4.3. Experimental Processes and Results

**Figure 8:**
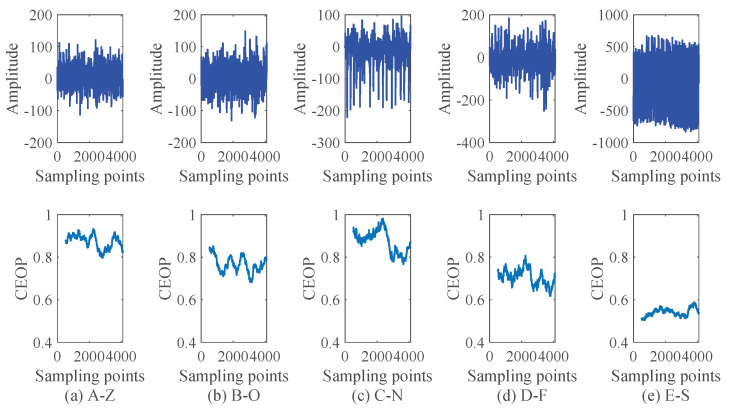
The original waveform and its corresponding values of the CEOP: (**a**) The waveform and the CEOP of A-Z. (**b**) The waveform and the CEOP of B-O. (**c**) The waveform and the CEOP of C-N. (**d**) The waveform and the CEOP of D-F. (**e**) The waveform and the CEOP of E-S.

**Figure 9:**
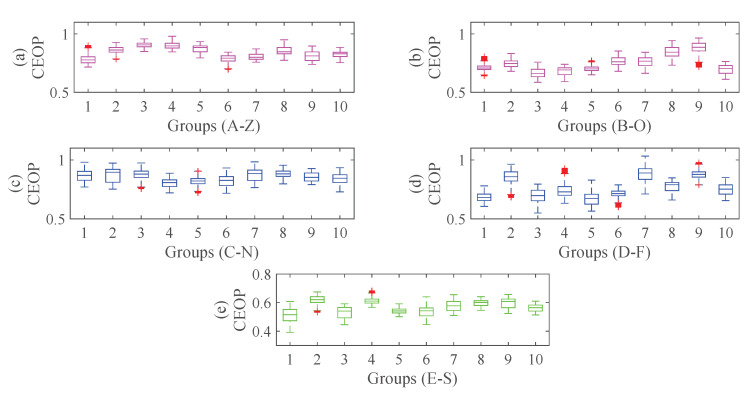
The boxplots of the distribution of the CEOP: (**a**) The CEOP of 10 groups of A-Z. (**b**) The CEOP of 10 groups of B-O. (**c**) The CEOP of 10 groups of C-N. (**d**) The CEOP of 10 groups of D-F. (**e**) The CEOP of 10 groups of E-S.

Table 1 summarizes details of the Bonn epilepsy EEG database. Each set contains 100 single-channel segments of 23.6 s duration from continuous EEG recordings, which are cut out after visual selection for artifacts. All of the EEG signals are band-pass filtered at 0.53–40 Hz [40]. We select all data sets to conduct the experiment to distinguish the epileptic and non-epileptic EEG signals. We extract the CEOP of all data sets with ordinal pattern order $d_{optimal}=3$ and time delay $\tau_{optimal}=1$ $s^{-1}$. For each set of the database, we randomly select a sample, whose wave and corresponding CEOP are shown in Figure 8. We can clearly see that the CEOP between the normal state (A-Z, B-O) and the inter-ictal state (C-N, D-F) are at a high level, which illustrates that there are low amplitude and high oscillation frequency, and the states are more complex. The epileptic spikes during seizures (E-S) is high amplitude and low oscillation frequency, thus its state is regular. To illustrate the difference of the features distribution better, we set up the boxplots by selecting ten group samples randomly for each set in Figure 9. The boxplots can visually judge the discrete distribution of the data and not be affected by outliers. We can clearly see that the CEOP in the ictal state is completely different from those in the other two states, which shows that it is meaningful to use the CEOP as a recognition metric.

The above analysis illustrates that the CEOP can be used as the feature vector to be input into the SVM classifier for recognition. Next, we identify the epileptic EEG signals that are based on the SVM. The recognition of epileptic activity (E-S) is performed against normal activity (A-Z and B-O) and inter-ictal activity (C-N and D-F), establishing four different binary classification problems. We mark A-Z, B-O, C-N, and D-F as the positive class, respectively, and E-S as a negative class in each experiment. We set the ratio of the training set to the testing set to be 8:2. In each binary classification experiment, the capacity of randomly selected training samples is 160 including 80 positive samples and 80 negative samples, and the capacity of testing samples is 40, including 20 positive samples and 20 negative samples.

**Figure 10:**
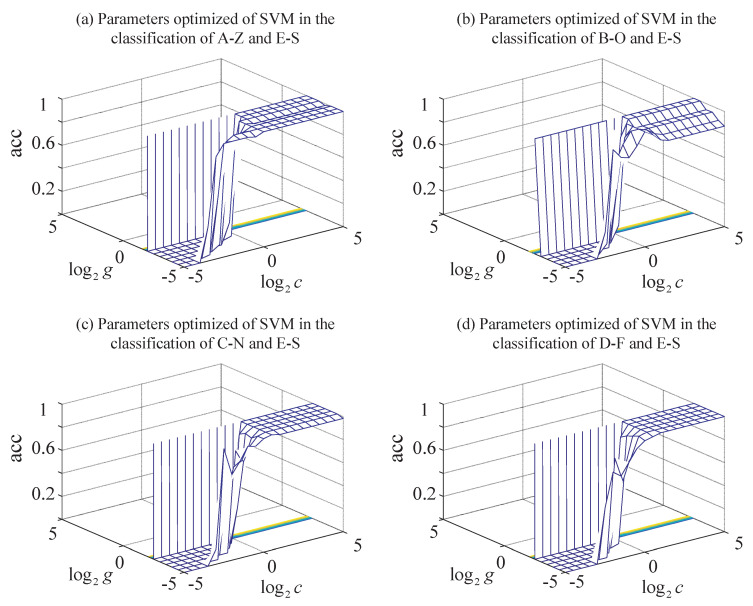
The contour map of the parameters optimized in the SVM classifier (3D view): (**a**) parameters optimized of SVM in the classification of A-Z and E-S. (**b**) Parameters optimized of SVM in the classification of B-O and E-S. (**c**) Parameters optimized of SVM in the classification of C-N and E-S. (**d**) Parameters optimized of SVM in the classification of D-F and E-S.

Grid search and 10-fold cross-validation are combined to determine the best parameter pair (c,g) in order to overcome the disadvantages of the over-learning and under-learning caused by parameters selection randomly in the SVM classifier. The contour map of the parameter pair (c,g) optimized in the SVM classifier (3D view) is in Figure 10. The parameter pair (c,g) corresponding to the point with the highest of the accuracy of the cross-validation acc is the best parameter pair. The best penalty factor $c_{b}$ and the best width parameter of the RBF $g_{b}$ are in Table 5.

**Figure 11:**
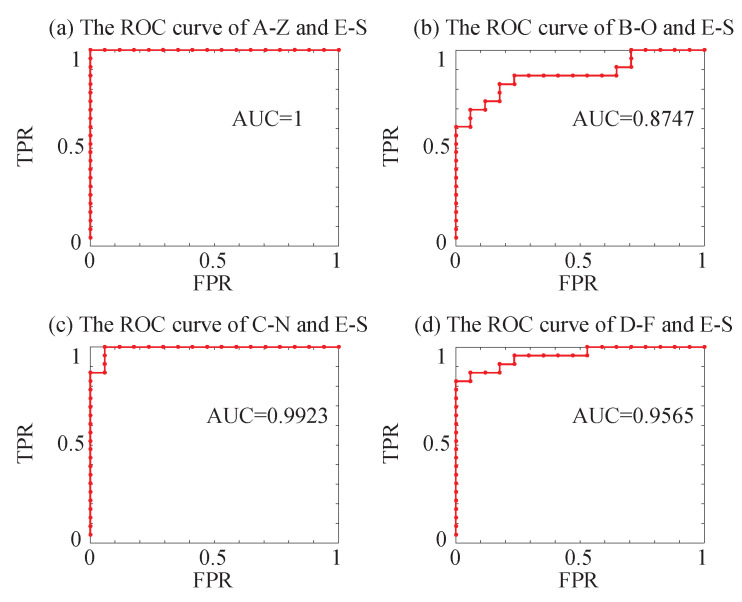
The ROC curves in the recognition of the epilepsy EEG signals: (**a**) The ROC curve of A-Z and E-S. (**b**) The ROC curve of B-O and E-S. (**c**) The ROC curve of C-N and E-S. (**d**) The ROC curve of D-F and E-S.

The optimized SVM classifier model is used to test the testing set after determining the optimal parameters of the SVM classifier. The sensitivity, specificity, accuracy and AUC are shown in Table 6. As we all know, when the value of AUC is 1, it is a perfect classifier. The sensitivity refers to the correct degree of the patient, that is, the percentage of the actual illness that is diagnosed correctly. The sensitivity of A-Z and E-S is best. The specificity is the degree that a non-patient is correctly identified, which is, the percentage of patients who are correctly diagnosed is being free of disease. The specificity of C-N and E-S is best. The accuracy refers to the result of the classification of testing set by optimized SVM classifier, and the recognition of A-Z and E-S has the best accuracy. Figure 11 shows the ROC curve. The curve is on the upper left of the diagonal, and the farther away it is, the better the classification is. The classification effect of A-Z and E-S is the best, followed by C-N and E-S, D-F, and E-S, and B-O and E-S. We will introduce the interpretation in the next section.

## 5. Discussions

The CEOP exhibits specific changing features for different brain states. The characteristic is consistent with the simulating brain rhythms (Figure 4) and the real EEG signals (Figure 8). The recognition experiments show that the results of A-Z and E-S have the best sensitivity, AUC, and accuracy, while the results of C-N and E-S have the best specificity. When considering all evaluation indexes comprehensively, the effect of the identification of B-O and E-S is the least obvious. This result is consistent with the result given in literature [36]. There are the largest overlaps of the CEOP of B-O and E-S, since B-O has lower CEOP as compared to other non-seizure sets. There is the fact that the EEG signals of B-O are obtained from five healthy awake volunteers with eyes closed, which causes the brain activity with more regular rhythms.

The Bonn epilepsy EEG database has been widely applied to evaluate the seizure recognition algorithms. The recognition problem of normal (A-Z) and ictal (E-S) EEG signals is most commonly used to compare the performance of different classification algorithms. The comparison between the CEOP combined with the SVM classifier that is proposed in the current work and other existing methods are provided in Table 7. It is obvious by comparison that increasing the number of extracted features will improve the recognition effect. In [17,18,41,42,43], all of them have achieved good classification accuracies with a variety of features extracted, and some of them go as high as 100% of accuracy. However, the algorithm complexity and calculation cost will increase with the increase of the number of features extracted. The whole process of feature extraction and classification will be redundant and complicated. In terms of selecting a single eigenvector, as compared with 93.55% of accuracy of the result given in literature [36], the accuracy of our approach is 95.00%. The distinguishing performance of the CEOP is better than that of the PE, which is consistent with our comparative performance analysis that is based on the neural mass model. This indicates that the CEOP as the single feature vector combined with the SVM classifier can effectively distinguish the epileptic EEG signals. As a kind of ordinal-patterns-based methods, the CEOP requires less prior knowledge regarding the time series. The idea of ordinal pattern analysis considers the sequence of ordinal patterns and the ordinal patterns distribution obtained instead of the original time series, thus the CEOP can measure the complexity of the EEG signals effectively and easily.

The Bonn epilepsy EEG database is mixed with scalp (A-Z and B-O) and intracranial (C-N, D-F and E-S) recordings. Thus, it might not be the perfect database to test various recognition algorithms due to the amplitudes of intracranial recordings and different locations of electrodes. In this work, the CEOP is as a single feature vector and it has achieved reliable results in the recognition of epilpesy EEG signals. However, as compared with multiple feature vectors, the CEOP still has certain limitations. The information extracted from a single feature is not as rich and detailed as the information extracted from multiple features. In addition, whether based on the neural mass model or the Bonn epilepsy EEG database, we only consider a one-dimensional time series. The application of the CEOP to the multichannel EEG signals is worthy of consideration and research. Increasing amounts of extracted features and classifiers are applied to the recognition of the epileptic EEG signals. We employ the CEOP as a eigenvector and combine it with the SVM classifier, which belongs to the traditional epileptic EEG signal classification strategy. The traditional epilepsy recognition method is to extract the features and then send them into the classifier. Features extraction mainly rely on artificial selection, which may lose some significant information of the original data. Some existing researches use sparse representation and dictionary learning to classify and detect epileptic EEG signals and they have achieved good classification results [15,44,45]. The classification of epileptic EEG signals based on sparse representation and its variants avoids tedious features extraction, and the algorithm runs fast, which is also worth studying in the future.

The comparison between the CEOP combined with the SVM classifier that is proposed in the current work and other existing methods are provided in Table 7.

## 6. Conclusions

The work mainly presents a novel recognition strategy for epilepsy EEG signals that is based on the conditional entropy of ordinal patterns. We discuss that the optimal parameters for the CEOP, including the ordinal pattern order $d_{optimal}=3$ and the time delay $\tau_{optimal}=1$ $s^{-1}$. The CEOP can accurately extract the complexity information of the time series, and the EEG signals in non-seizures and seizures are effectively distinguished. Subsequently, we apply the CEOP combined with SVM classifier to the real Bonn epilepsy database, and the results verify that the CEOP has good recognition performance. In terms of EEG classification using the CEOP as a single feature vector, its AUC is almost 1. This shows that the method is feasible in the distinction of epileptic EEG signals, and it can provide a strong basis for the judgment of clinical epileptic signals in each period. In the future, we will try to combine the CEOP with other classification algorithms and nonlinear measurement indexes to form a compound recognition strategy with multiple eigenvectors. 

## Figures and Tables

**Table 1:** Summary of the Bonn epilepsy electroencephalogram (EEG) database.

	Data Sets	A-Z	B-O	C-N	D-F	E-S
Category						
**Experimental subject**		Five healthy volunteers		Five epilepsy patients		
**EEG type**		Scalp	Scalp	Intracranial	Intracranial	Intracranial
		EEG	EEG	EEG	EEG	EEG
**Subject status**		Awake,	Awake,	Inter-ictal	Inter-ictal	Ictal
		eyes open	eyes closed	stage	stage	stage
**Electrode placement**		International	International	Hippocampus	Within	Within
		10-20	10-20	opposite to	epileptogenic	epileptogenic
		system	system	hemisphere	zone	zone
**Number of subsets**		100	100	100	100	100
**Sampling points**		4097	4097	4097	4097	4097
**Sampling frequency**		173.61Hz	173.61Hz	173.61Hz	173.61Hz	173.61Hz

**Table 2:** Some concepts between the true values and predicted values.

Category	True Value	Predicted Value
**TP**	positive	positive
**FP**	negative	positive
**TN**	negative	negative
**FN**	positive	negative

**Table 3:** Statistical values of the permutation entropy (PE), modified permutation entropy (MPE), and CEOP under different excitability gain parameter *A*.

Category	*A* (mV)	Mean			Std		
		PE	MPE	CEOP	PE	MPE	CEOP
**Normal**	**3.25**	0.6124	0.6164	0.5590	0.0319	0.0230	0.0468
	**3.6**	0.2628	0.2686	0.1031	0.0302	0.0133	0.0205
	**3.8**	0.2590	0.2604	0.0872	0.0135	0.0111	0.0098
	**4.0**	0.2660	0.2675	0.0913	0.0064	0.0053	0.0062
**Seizure**	**4.2**	0.2722	0.2741	0.0973	0.0132	0.0102	0.0093
	**4.4**	0.2780	0.2804	0.1021	0.0164	0.0083	0.0114
	**4.6**	0.2810	0.2836	0.1055	0.0172	0.0042	0.0120
	**4.8**	0.2851	0.2879	0.1087	0.0167	0.0021	0.0120

**Table 4:** Statistical values of the PE, MPE, and CEOP under different input Gaussian white noise p(t).

Category	$p_{\mathrm{avg}}$	Mean			Std		
		PE	MPE	CEOP	PE	MPE	CEOP
	**81**	0.6143	0.6184	0.5600	0.0336	0.0234	0.0510
	**91**	0.6109	0.6150	0.5572	0.0297	0.0207	0.0432
**Normal (** *A* **= 3.25 mV)**	**101**	0.6089	0.6128	0.5572	0.0327	0.0224	0.0508
	**111**	0.6142	0.6187	0.5636	0.0356	0.0207	0.0514
	**121**	0.3455	0.3505	0.1877	0.0396	0.0281	0.0468
	**81**	0.4245	0.4301	0.3109	0.0565	0.0361	0.0705
	**91**	0.2578	0.2626	0.0994	0.0305	0.0129	0.0200
**Seizure (** *A* **= 3.8 mV)**	**101**	0.2569	0.2585	0.0857	0.0146	0.0116	0.0102
	**111**	0.2690	0.2703	0.0916	0.0053	0.0042	0.0052
	**121**	0.2777	0.2793	0.0973	0.0120	0.0095	0.0088

**Table 5:** The best parameters of the SVM classifier and the accuracy of cross-validation.

	Classification	A-Z, E-S	B-O, E-S	C-N, E-S	D-F, E-S
Category					
$c_{b}$		1	1	1.4142	2
$g_{b}$		0.0313	0.0313	0.0313	0.0313
$acc_{b}$ (%)		96.25	81.25	90.63	88.75

**Table 6:** The performance of the recognition represented by four evaluation indexes.

	Classification	A-Z, E-S	B-O, E-S	C-N, E-S	D-F, E-S
Evaluation Index					
**Sensitivity (%)**		100	80.00	88.46	86.96
**Specificity (%)**		89.47	80.00	100	82.35
**Accuracy (%)**		95.00	80.00	92.50	85.00
**AUC**		1	0.8747	0.9923	0.9565

**Table 7:** The comparison on the recognition of epilepsy EEG signals of A-Z and E-S between existing approaches.

Authors	Method (Features Extraction & Classifier)	Number of Extracted Features	Accuracy (%)
Kannathal et al. [46]	Entropy measures &	4	92.22
(2005)	Adaptive neuro-fuzzy inference system (ANFIS)
Subasi [47]	Discrete wavelet transform (DWT) &	16	94.50
(2007)	Mixture of experts (ME)
Iscan et al. [41]	Cross correlation (CC), power spectral density (PSD) &	2	100
(2011)	Least squares support vector machine (LS-SVM)
Nicolaou et al. [36]	Permutation entropy (PE) &	1	93.55
(2012)	Support vector machine (SVM)
Fu et al. [17]	Hilbert marginal spectrum analysis (HMS) &	8	99.85
(2015)	Support vector machine (SVM)
Swami et al. [42]	Dual-tree complex wavelet transform (DTCWT) &	6	100
(2016)	General regression neural network (GRNN)
Deriche et al. [18]	Singular value decomposition (SVD) &	2	99.30
(2019)	Multilayer perceptron network (MLP)
Zhou et al. [43]	Wave coefficients, entropy measures &	4	96.30
(2020)	Improved convolution neural network (CNN)
This work	Conditional entropy of ordinal patterns (CEOP) &	1	95.00
	Support vector machine (SVM)		
